# Real-World Data on the Safety and Efficacy of SBRT for Central and Ultra-Central Lung Tumors: A Retrospective Multi-Center Cohort

**DOI:** 10.3390/cancers18040653

**Published:** 2026-02-17

**Authors:** Anna Zygogianni, Andromachi Kougioumtzopoulou, Kalliopi Platoni, Maria Protopapa, Zoi Liakouli, Ioannis M. Koukourakis, Despoina Alexiou, Theodoros Stroubinis, Christina Armpilia, Christos Antypas, Michalis Psarras, Despoina Stasinou, Ioannis Georgakopoulos, Vasileios Kouloulias

**Affiliations:** 1 Department of Clinical Radiation Oncology, Medical School, National and Kapodistrian University of Athens, “Attikon” University Hospital, 12462 Haidari, Greece; azygogianni@med.uoa.gr (A.Z.); ikoukourakis@med.uoa.gr (I.M.K.);; 2 Center of Radiation Oncology and Stereotactic Radiosurgery, “Mediterraneo” General Hospital, 16675 Glyfada, Greece; 3 Laboratory of Applied Medical Physics, Medical School, National and Kapodistrian University of Athens, “Attikon” University Hospital, 12462 Haidari, Greece; 4 1st Department of Radiology, National and Kapodistrian University of Athens, “Aretaieion” University Hospital, 11528 Athens, Greececantypas@med.uoa.gr (C.A.);

**Keywords:** stereotactic ablative radiotherapy, stereotactic body radiotherapy, non–small cell lung cancer, central lung tumors, ultra-central lung tumors, early-stage lung cancer, organ-at-risk toxicity, real-world data, local control

## Abstract

**Background/Objectives**: The use of stereotactic body radiotherapy (SBRT) for centrally and ultra-centrally located early-stage non-small cell lung cancer (NSCLC) remains clinically challenging due to the proximity of critical mediastinal organs at risk and the limited prospective evidence, particularly for ultra-central disease. Real-world data are needed to better define the safety and efficacy of SBRT when delivered according to contemporary protocol-based planning principles. **Methods**: This retrospective cohort study included patients treated at two radiotherapy centers, with centralized follow-up and outcome adjudication at a single academic institution. We evaluated patients with centrally or ultra-centrally located, early-stage NSCLC treated with SBRT according to dose-fractionation and planning principles derived from the NRG Oncology/RTOG 0813 protocol. Tumors were classified as central or ultra-central according to the International Association for the Study of Lung Cancer and HILUS definitions, respectively. The prescribed dose was 50 Gy in five fractions. Primary endpoints were treatment-related toxicity and local progression-free survival (LPFS). Secondary endpoints included overall survival (OS), progression-free survival (PFS), and dosimetric outcomes. Survival endpoints were analyzed using the Kaplan–Meier method. **Results**: Seventy-eight patients were included, of whom 52 had centrally located and 26 ultra-centrally located tumors. Median follow-up was 57 months. The overall objective response rate was 92.3%. The estimated 4-year LPFS was 97.4% for the entire cohort, with rates of 100% for central and 91.6% for ultra-central tumors (*p* < 0.001). Four-year OS was 98.7%, with no treatment-related deaths observed. Treatment-related toxicity was minimal, with grade ≥ 2 events occurring in only one patient (1.3%) and no grade ≥ 3 toxicity. All treatment plans met predefined organ-at-risk dose constraints. **Conclusions**: In this real-world cohort, SBRT delivered according to RTOG 0813 planning principles achieved excellent local control with minimal clinically relevant toxicity in patients with centrally and ultra-centrally located early-stage NSCLC. These findings support the safe implementation of SBRT in carefully selected patients when stringent organ-at-risk constraints and conservative treatment planning are employed.

## Simple Summary

Stereotactic body radiotherapy (SBRT) is an effective treatment for patients with early-stage lung cancer who are not suitable for surgery. However, when lung tumors are located close to critical structures such as the airways, heart, or major blood vessels (so-called central and ultra-central tumors), treatment is more challenging and safety concerns remain. In this study, we evaluated the real-world outcomes of SBRT in patients with centrally and ultra-centrally located early-stage non–small cell lung cancer treated according to established planning principles. We found that SBRT achieved excellent local tumor control with very low rates of clinically relevant side effects, even for ultra-central tumors. Importantly, no severe or treatment-related deaths were observed. These findings suggest that, when careful treatment planning and strict protection of nearby organs are applied, SBRT can be safely used for selected patients with central and ultra-central lung tumors in routine clinical practice.

## 1. Introduction

Stereotactic body radiotherapy (SBRT) is an established curative-intent treatment for patients with early-stage peripheral non–small cell lung cancer (NSCLC) who are medically inoperable or decline surgical resection [1,2,3,4,5]. Compared with no active treatment, the implementation of SBRT in patients with localized T1–2N0M0 NSCLC confers a substantial survival benefit, increasing 5-year relative survival from 7% to 44% and extending median overall survival (OS) from 11 to 47 months [6,7]. Randomized and prospective comparative studies suggest that SBRT may offer improved local control and comparable or favorable survival outcomes compared with conventionally fractionated radiotherapy in medically inoperable early-stage NSCLC [8,9,10].

In contrast to peripheral disease, the application of SBRT for centrally and ultra-centrally located lung lesions remains clinically challenging, due to the proximity of critical mediastinal organs at risk (OARs). Early-phase studies reported excessive treatment-related toxicity when SBRT was delivered using highly hypofractionated regimens in central tumors; however, subsequent trials incorporating dose reduction and more fractionated schedules demonstrated that SBRT can be delivered safely when stringent OAR constraints are applied [11,12]. Centrally located tumors are generally defined according to established criteria, such as the International Association for the Study of Lung Cancer (IASLC) definition, whereas ultra-central tumors lack a universally accepted definition and show substantial heterogeneity across studies and clinical protocols [3,13,14].

Prospective evidence supporting SBRT for centrally located, inoperable early-stage NSCLC derives from several phase I/II and phase II trials and multi-institutional experiences, demonstrating favorable tumor control with an acceptable though non-negligible risk of severe toxicity when appropriately fractionated regimens and strict OAR constraints are employed [12,15,16,17]. In contrast, evidence for SBRT in ultra-central lung tumors remains limited, with most data originating from retrospective series [13,18,19,20]. To date, the HILUS and SUNSET trials represent the only dedicated prospective studies in this setting, while NRG Oncology/RTOG 0813 included only a limited subset of patients with ultra-central characteristics [12,21,22]. Consequently, clinical guideline recommendations for ultra-central lung tumors have emerged only recently, largely based on expert consensus [13].

Therefore, real-world data are needed to better define the safety and efficacy of SBRT in patients with centrally and ultra-centrally located lung tumors treated in routine clinical practice. The present study aimed to evaluate treatment-related toxicity and clinical outcomes of SBRT in a real-world, multi-center cohort of patients with centrally and ultra-centrally localized early-stage NSCLC treated according to dose-fractionation and planning principles derived from the NRG Oncology/RTOG 0813 protocol.

## 2. Materials and Methods

### 2.1. Study Design

This is a retrospective cohort study investigating the safety and efficacy of SBRT in patients with centrally or ultra-centrally localized NSCLC. Treatment delivery occurred at two radiotherapy centers, while clinical follow-up, data collection, and outcome adjudication were performed centrally at the Department of Clinical Radiation Oncology, “ATTIKON” University Hospital. Patients treated between 26 October 2020 and 5 February 2024 were screened for eligibility, and all consecutively treated eligible patients during this period were included in the analysis. Follow-up was defined as the time from the date of the first SBRT fraction and was censored at the predefined data cutoff date of 1 January 2026. The study was approved by the Ethical and Scientific Committee of the local institution.

### 2.2. Patient Selection

All included patients had lesions classified as either central or ultra-central in location. Tumors were classified as central according to the IASLC definition, defined as a gross tumor volume (GTV) located within a 2 cm zone around any mediastinal critical structure, including the bronchial tree, major vessels, heart, esophagus, spinal cord, phrenic nerve, recurrent laryngeal nerve, and brachial plexus [3]. Ultra-central lesions were defined as tumors with proximity within 1 cm of the trachea or mainstem bronchus based on the HILUS phase II study [21]. Because this was a retrospective multi-center study with centralized follow-up, detailed geometric information regarding direct planning target volume (PTV) overlap with the proximal bronchial tree or trachea was not systematically available.

Eligible patients had histologically confirmed NSCLC whenever feasible; in cases where biopsy was contraindicated or not possible, diagnosis was established based on multidisciplinary tumor board consensus using radiological criteria and FDG positron emission tomography scan (PET/CT) findings. Tumors had a maximum diameter ≤ 3 cm and were staged as clinical T1N0M0 according to the 8th edition of the AJCC Cancer Staging System [23]. Baseline thoracic imaging via a computed tomography (CT) scan and functional staging utilizing PET/CT were performed in all patients. Only individuals suspected of mediastinal lymph node involvement, as indicated by CT or PET/CT, were sampled to verify the N0 status.

Patients were included for SBRT treatment if they were either medically inoperable by a multidisciplinary tumor board or had declined surgical resection.

Exclusion criteria included prior thoracic RT to the target lesion, the presence of multiple synchronous lung lesions, or recurrent lung cancer. Patients with histology other than NSCLC, insufficient imaging or pathological confirmation, or missing follow-up data were excluded.

### 2.3. Radiotherapy Planning and Delivery

The prescribed regimen of 50 Gy in five fractions reflected routine clinical practice during the study period and was determined by the treating radiation oncologist based on tumor location, proximity to critical structures, and the ability to meet organ-at-risk constraints derived from the NRG Oncology/RTOG 0813 protocol [12]. Patients were immobilized in the supine position using a customized immobilization device to minimize setup variability. Four-dimensional CT (4D-CT) was performed in all patients to account for respiratory motion.

GTV was delineated on lung windows settings, with reference to diagnostic images and PET/CT. No clinical target volume (CTV) expansion was applied for microscopic disease. An internal target volume (ITV) was generated to encompass the tumor motion across all respiratory phases derived from the 4D-CT dataset for all patients. PTV was created by applying an isotropic margin of 5mm to the ITV and then modified according to the adjacent normal anatomic structures. Patients were treated either on a Varian EDGE linear accelerator platform (Varian Medical Systems, Palo Alto, CA, USA) or CyberKnife M6 robotic radiosurgery system (Accuray Inc., Madison, WI, USA). Varian EDGE treatment was delivered using image-guided radiotherapy (IGRT) with cone-beam CT (CBCT), while the surface-guided radiotherapy (SGRT) was performed with the AlignRT system (ver. 6.3.247.2). The treatment planning system used the ECLIPSE VARIAN ver. 15.6. Cyberknife was equipped with an InCise Multileaf Collimator and with a lung-optimized treatment (LOT) module, together with the real-time image-guided Synchrony Respiratory Tracking System (Accuray, Inc.) to deal with target movement. Accuray Precision Treatment Planning software (v.3.1.1.1) was used to generate treatment plans for the CyberKnife.

The SBRT regimen consisted of five daily fractions of 10 Gy each. The prescribed total dose of 50 Gy in 5 fractions corresponded to a biologically effective dose (BED) of 100 Gy for tumor tissue (α/β = 10 Gy) and 216.16 Gy for late-responding normal tissues (α/β = 3 Gy).

The dose was prescribed to the PTV in accordance with the RTOG 0813 framework. In detail, the following SBRT principles were applied, including high conformality and appropriate selection of the prescription isodose line (typically 60–90% of the maximum dose at the center of the target). The prescription was selected such that at least 99% of the PTV received a minimum of 90% of the prescribed dose, while hot spots within organs at risk (OARs) were strictly avoided. The organ-at-risk (OAR) constraints were prioritized over target coverage, particularly for critical mediastinal structures. Dose compromise was permitted when standard PTV coverage could not be achieved without exceeding dose OAR constraints. Dose–volume constraints for the proximal bronchial tree, trachea, esophagus, spinal cord, heart, and lungs were in line with the RTOG 0813 recommendations (Table 1).

### 2.4. Follow-Up and Response Assessment

All patients were followed up at “ATTIKON” University Hospital of Athens according to institutional protocol after completion of SBRT. Clinical evaluation and thoracic CT imaging were performed every 3–4 months during the first two years and every 6 months thereafter. PET/CT was performed when clinically indicated, particularly in cases of equivocal CT findings or suspicion of disease recurrence. Follow-up was defined as the interval from the first SBRT fraction to the date of last clinical contact or imaging assessment. Median follow-up time was estimated using the reverse Kaplan–Meier method. Follow-up was censored at the predefined data cutoff date of 1 January 2026.

Tumor response and disease progression were assessed based on serial imaging and classified according to the Response Evaluation Criteria in Solid Tumors (RECIST), version 1.1 [24]. Local failure was defined as radiographic progression within the PTV, with histological confirmation obtained when feasible. In cases of suspected local progression where imaging findings were equivocal, biopsy was pursued when clinically feasible and when the result was expected to influence management. Histological confirmation was obtained in a subset of cases, and when post-treatment fibrosis or benign changes were demonstrated, these cases were not counted as true progression events. When biopsy was not feasible due to comorbidities, procedural risk, or patient preference, outcomes were adjudicated by multidisciplinary consensus based on serial imaging and clinical follow-up.

Treatment-related toxicity was assessed during treatment and at each follow-up visit and graded according to the Common Terminology Criteria for Adverse Events (CTCAE), version 5.0 [25]. Acute and late radiation-related toxicities were defined as events occurring within or beyond 90 days from SBRT completion, respectively.

### 2.5. Endpoints

The primary endpoints of the study were treatment-related toxicity stratified by tumor location (central versus ultra-central) and local progression-free survival (LPFS).

LPFS was used as a surrogate for local control and was defined as the time from the date of the first SBRT fraction to radiographic local tumor progression within the irradiated planning target volume (PTV) or death from any cause, whichever occurred first.

Secondary endpoints included overall survival (OS), progression-free survival (PFS), and dosimetric analysis of OARs, stratified by tumor location (central versus ultra-central). Overall survival (OS) was calculated from the date of the first SBRT fraction to the date of death from any cause. Progression-free survival (PFS) was defined as the time from the date of the first SBRT fraction to the first occurrence of local, regional, or distant disease progression, or death from any cause, whichever occurred first.

### 2.6. Statistical Analysis

Descriptive statistics were used to summarize clinical data. Continuous variables were reported as mean ± standard deviation or median (range), and categorical variables as frequencies and percentages. Age was summarized for the overall cohort and by tumor location; no additional sex-stratified age analyses were performed. Comparisons between central and ultra-central tumors were performed using the Wilcoxon rank-sum test or Student’s *t*-test for continuous variables and the chi-squared or Fisher’s exact test for categorical variables; performance status was analyzed as PS 0 vs. PS ≥ 1.

Time-to-event endpoints (LPFS, PFS, OS) were calculated from the date of the first SBRT fraction and were analyzed using the Kaplan–Meier method, with group comparisons performed using the log-rank test. Treatment-related toxicity was summarized descriptively, with clinically relevant toxicity defined as grade ≥ 2. Because of the limited number of outcome events, multivariable analyses and regression-based effect size estimates (e.g., hazard ratios) were not considered statistically reliable and were therefore not performed. Survival comparisons are therefore presented descriptively using Kaplan–Meier curves and log-rank testing. All tests were two-sided, with *p* < 0.05 considered statistically significant, and analyses were performed using standard statistical software. Subgroup comparisons were considered exploratory; no formal adjustment for multiple comparisons was applied.

## 3. Results

A total of 78 patients met the eligibility criteria and were treated between 26 October 2020 and 5 February 2024; 52 patients had centrally located tumors, and 26 had ultra-central tumors. Follow-up was censored at the predefined data cutoff date of 1 January 2026. The maximum observed follow-up was 58 months. The median follow-up, estimated using the reverse Kaplan–Meier method, was 57 months (IQR 53–58) for the overall cohort, 58 months (IQR 56.8–58) for patients with central tumors, and 53 months (IQR 48–54) for patients with ultra-central tumors.

### 3.1. Patient and Tumor Characteristics

Baseline patient and tumor characteristics are summarized in Table 2. The median age of the cohort was 76.0 years (IQR 69.0–80.0) and did not differ significantly between groups (*p* = 0.146). Male sex was more frequent among patients with central tumors (*p* = 0.008). ECOG performance status differed between groups, with a higher proportion of ECOG PS 0 in the ultra-central cohort (*p* = 0.022).

Histological distribution differed significantly (*p* = 0.028), with squamous cell carcinoma more frequent among ultra-central tumors, whereas cases without histological confirmation were observed only in the central cohort. Median tumor diameter did not differ significantly between groups (*p* = 0.307); however, ultra-central tumors had significantly smaller tumor volumes (*p* = 0.004). Most lesions were staged as T1a–T1b (≤2 cm), with no significant differences in cT-stage or lobar location between groups.

### 3.2. Treatment Response and Local Control

Radiological response was assessable in all patients and is detailed in Table 3. The overall objective response rate (ORR) was 92.3%, comprising 38 complete and 34 partial responses. Stable disease was observed in one patient and progressive disease in five patients.

Response rates were comparable between central and ultra-central tumors (ORR 92.3% in both groups). In four patients with central tumors initially classified as progressive disease, histological reassessment confirmed post-treatment fibrosis without viable tumor; these cases were not considered progression events.

**Figure 1:**
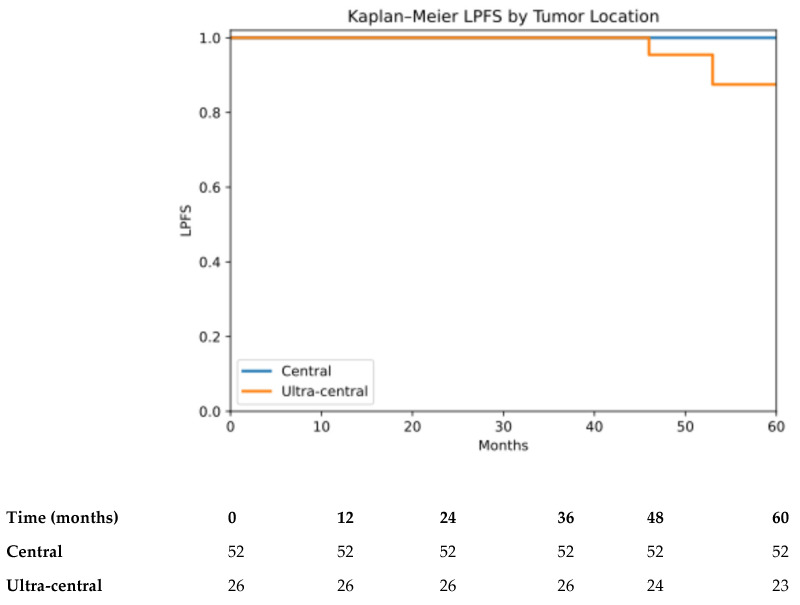
LPFS by tumor location (central vs. ultra-central). Kaplan–Meier estimates of LPFS stratified by tumor location, central vs. ultra-central (log-rank *p* < 0.001). LPFS was calculated from the date of the first SBRT fraction.to radiographic local tumor progression within the irradiated planning target volume or death from any cause. Numbers at risk at selected time points are shown below the x-axis.

LPFS was analyzed from the date of the first SBRT fraction. Seventeen LPFS events occurred during follow-up. The estimated 4-year LPFS rate was 97.4% (95% CI: 89.9–99.3) for the entire cohort (Supplementary Figure S1). When stratified by tumor location, 4-year LPFS was 100% for central tumors and 91.6% (95% CI: 70.1–97.8) for ultra-central tumors (log-rank *p* < 0.001) (Figure 1).

### 3.3. PFS and OS

**Figure 2:**
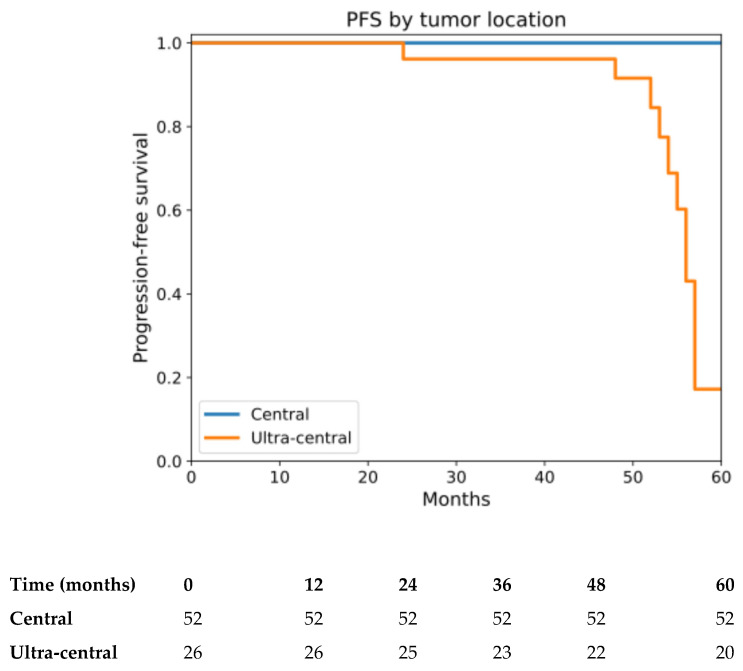
PFS by tumor location (central vs. ultra-central). Kaplan–Meier estimates of PFS stratified by tumor location (central vs. ultra-central), calculated from the date of the first SBRT fraction. (log-rank *p* < 0.001). An event was defined as disease progression (local, regional, or distant) or death from any cause. Radiographic progression that was histologically reassessed and demonstrated no viable tumor was not considered a progression event. Analyses were restricted to the first 60 months of follow-up, in accordance with the observed follow-up duration. Numbers at risk at selected time points are shown below the x-axis.

PFS was analyzed up to 60 months in accordance with observed follow-up duration. Eleven PFS events were recorded, all occurring in patients with ultra-central tumors. Median PFS was not reached. The estimated 4-year PFS rate was 97.4% (95% CI: 89.9–99.3) for the overall cohort (Supplementary Figure S2), with rates of 100% for central tumors and 91.6% (95% CI: 70.1–97.8) for ultra-central tumors (log-rank *p* < 0.001) (Figure 2). Radiographic progression in four patients with central tumors was histologically reassessed with repeat biopsy and demonstrated no evidence of viable tumor, with findings consistent with post-treatment fibrosis; these cases were therefore not considered progression events in the PFS analysis.

**Figure 3:**
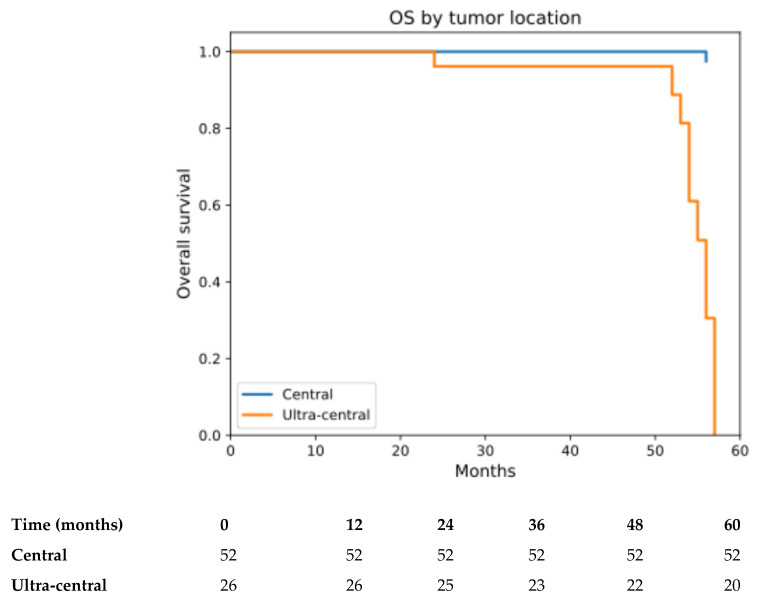
OS by tumor location (central vs. ultracentral). Kaplan–Meier estimates of OS stratified by tumor location (central vs. ultra-central), calculated from the date of the first SBRT fraction to death from any cause (log-rank *p* < 0.001). Analyses were restricted to the first 60 months of follow-up, in accordance with the observed follow-up duration. Survival outcomes differed significantly between groups, with inferior OS observed in patients with ultra-central tumors (log-rank, *p* < 0.001). Numbers at risk at selected time points are shown below the x-axis.

OS was analyzed from the date of the first SBRT fraction. At the time of analysis, 12 deaths had occurred, and the median OS had not reached. The estimated 4-year OS rate was 98.7% (95% CI: 92.1–99.8) for the overall cohort (Supplementary Figure S3). Four-year OS was 100% for central tumors and 96.2% (95% CI: 75.7–99.4%) for ultra-central tumors (log-rank *p* < 0.001) (Figure 3). Importantly, none of the recorded deaths were classified as treatment-related. Two patients died due to acute myocardial infarction, while the remaining deaths occurred in the context of disease progression, significant comorbidities, and advanced age, without clinical or radiographic evidence of SBRT-related toxicity.

### 3.4. Toxicity

Treatment-related toxicity was generally mild and summarized in Table 4. The majority of patients (89.7%) experienced no toxicity (grade 0). Any-grade toxicity (grade ≥ 1) occurred in 10.3% of patients and was predominantly grade 1. Clinically relevant toxicity (grade ≥ 2) was observed in one patient (1.3%), and no grade ≥ 3 toxicities were reported. Accordingly, the rate of grade ≥ 3 toxicity was 0/78 in the overall cohort (0.0%; 95% CI: 0.0–4.6%) and 0/26 in the ultra-central subgroup (0.0%; 95% CI: 0.0–13.2%).

When stratified by tumor location, no grade ≥ 2 toxicity occurred in patients with central tumors, whereas one patient with an ultra-central tumor experienced grade ≥ 2 toxicity (3.8%). No clinically significant airway toxicity, hemoptysis, major vascular events, or grade ≥ 2 radiation pneumonitis were observed in either group. Late toxicity was uncommon and observed exclusively in the ultra-central cohort. These events included one case of grade 1 esophagitis and two cases of dysphagia (one grade 1 and one grade 2). All events were managed conservatively and resolved without long-term sequelae.

### 3.5. Dosimetric Outcomes

All treatment plans met predefined OAR dose constraints as presented in Table 5. Dose exposure to critical mediastinal structures, including the esophagus, trachea, proximal bronchial tree, heart, great vessels, and spinal cord, remained consistently below protocol-specified limits. Lung dose constraints were respected across the cohort, with preserved lung volumes receiving ≤13.5 Gy for at least 1000 cc and ≤12.5 Gy for at least 1500 cc.

## 4. Discussion

In this real-world, multi-center cohort with centralized follow-up, SBRT delivered according to dose-fractionation and planning principles derived from the NRG Oncology/RTOG 0813 protocol achieved excellent local tumor control with a very low incidence of clinically relevant toxicity, including in patients with ultra-central tumors. With a median follow-up exceeding four years, the present study provides mature outcome data in a clinical setting where prospective evidence remains limited, particularly for ultra-central disease.

For centrally located tumors, our findings are consistent with prospective phase I/II and phase II studies, including NRG Oncology/RTOG 0813 and the EORTC 22113–08113 LungTech trial, which demonstrated that SBRT can be safely delivered when appropriately fractionated regimens and stringent OAR constraints are applied [12,15]. The absence of grade ≥ 3 toxicity and the very low rate of grade ≥ 2 events observed in our cohort compares favorably with these trials. This favorable toxicity profile likely indicates rigorous compliance with protocol-based organ-at-risk constraints, conservative treatment planning, and careful patient selection in routine clinical practice. The features of the tumor may have also influenced the observed safety outcomes.

Tumor characteristics may also have contributed to the observed safety outcomes. In our cohort, the majority of lesions were small, with a median maximum diameter of 17 mm and a median tumor volume of 23.2 cc. This aligns with other prospective experiences such as the LungTech trial, which predominantly enrolled smaller central tumors, whereas RTOG 0813 permitted a broader range of tumor sizes up to 5 cm. Differences in treated tumor or GTV size across studies may partly account for the variability in reported toxicity rates.

Importantly, all treatment plans in the present study achieved dosimetric parameters consistently within protocol-derived OAR constraints. Dose exposure to critical mediastinal structures, including the esophagus, proximal bronchial tree, trachea, heart, and great vessels, all remained well below established tolerance thresholds, even in ultra-central cases. Current evidence is limited and insufficient to guide definitive dosimetric decision-making. While some studies have suggested an association between high proximal bronchial tree doses and toxicity, these findings have been inconsistent, and pooled analyses have not demonstrated a clear dose–toxicity relationship [13,26,27,28]. Heterogeneous target and OAR definitions, limited dosimetric reporting, and variability in planning approaches further limit the applicability of existing data to routine clinical practice.

Evidence for SBRT in ultra-central lung tumors remains considerably more limited and is largely retrospective. Prospective data are confined to the HILUS and SUNSET trials, with only a small subset of ultra-central cases included in RTOG 0813 [12,21,22]. Comparison with the HILUS trial is particularly informative, as the present study and HILUS employed the same proximity-based definition of ultra-central disease, defined as lesions located within ≤1 cm of the trachea or main bronchi [21]. Despite this shared definition, important differences exist. Lesions in HILUS were larger and more heterogeneous in size and volume, and the cohort included both primary lung cancers and metastatic lesions [21]. By contrast, ultra-central tumors in the present study were smaller, more homogeneous, and limited to early-stage NSCLC, factors that may partly account for the lower incidence of clinically relevant toxicity observed.

Further insight is provided by the SUNSET trial, which adopted a more restrictive overlap-based definition of ultra-central disease, requiring direct overlap between the planning target volume and the proximal bronchial tree or trachea [22]. This definition identifies a particularly high-risk subgroup and results in larger treated volumes. In SUNSET, despite the use of fractionated regimens, a non-negligible incidence of grade ≥ 3 toxicity, including treatment-related deaths, was reported. In contrast, ultra-central tumors in the present cohort were defined by proximity rather than overlap, were smaller in size, and were treated following the RTOG 0813 framework for OAR constraints. Importantly, this distinction between proximity-based and overlap-based ultra-central definitions has major implications for toxicity risk, and our findings should therefore be interpreted as applicable primarily to proximity-defined ultra-central tumors rather than overlap-defined, higher-risk cases. A comparison of central and ultra-central definitions across key studies is provided in Supplementary Table S1. Because overlap-based geometric information was not systematically recorded, we cannot determine the extent to which any lesions met overlap-defined ultra-central criteria, and our findings should therefore be interpreted primarily within the framework of proximity-defined ultra-central disease. These differences likely contributed to the favorable toxicity profile observed and highlight the importance of careful patient selection and treatment planning. Therefore, our findings should be interpreted as applicable to carefully selected ultra-central tumors defined by proximity criteria and meeting strict organ-at-risk constraints, rather than to overlap-defined or higher-risk ultra-central tumors as studied in SUNSET.

Although the RTOG 0813 trial explored dose levels up to 60 Gy in five fractions, all patients in the present cohort received 50 Gy in five fractions, reflecting physician-directed routine clinical practice during the study period within an RTOG 0813–based planning framework prioritizing organ-at-risk protection. More protracted fractionation schedules (e.g., 8–10 fractions) are increasingly adopted in overlap-defined ultra-central tumors in contemporary practice because of their higher baseline toxicity risk and difficulty in meeting organ-at-risk constraints [13]. The modestly lower LPFS observed in the ultra-central subgroup compared with central tumors cannot be attributed to dose selection alone in this retrospective dataset and may also reflect differences in tumor location, baseline characteristics, and limited event numbers. These subgroup differences should be interpreted as descriptive and hypothesis-generating, as tumor location alone cannot be considered an independent prognostic factor in the absence of multivariable adjustment. The optimal fractionation strategy for ultra-central disease remains an area of active investigation. Additionally, treatment planning in the present study consistently prioritized adherence to organ-at-risk constraints, with dose compromise accepted when necessary to protect critical mediastinal structures, which may have further contributed to the favorable safety profile observed.

The favorable outcomes observed in our cohort should be interpreted in the context of patient characteristics and follow-up practices. The median age was higher than in several prospective studies, while performance status was similar, suggesting that demographic factors alone do not explain the results and that competing non-cancer mortality may influence survival in this elderly population. Our patients had early-stage disease with generally small tumor volumes and were carefully selected and treated at experienced centers with centralized follow-up. The excellent local control, particularly the 100% 4-year LPFS in central tumors, likely reflects this selection, uniform delivery of a high biologically effective dose (50 Gy in 5 fractions), and strict adherence to protocol-based planning principles. These favorable survival outcomes likely reflect a combination of careful patient selection, smaller tumor burden, proximity-based ultra-central definition, and conservative constraint-driven planning, and therefore should not be considered broadly generalizable beyond similarly selected patients, particularly not to higher-risk or overlap-defined ultra-central tumors.

Numerous ongoing prospective studies aim to further refine the role of SBRT in NSCLC dedicated to central and ultra-central lesions via advanced image guidance, adaptive strategies, and combination approaches. The STRICT/STAR-LUNG trial (NCT05354596) is an open-label phase II study evaluating toxicity and local control in parallel cohorts of central standard linear accelerators using CBCT-guided adaptive SBRT and ultra-central lesions treated on MR-linac platforms with MR-guided online adaptation [29]. The MAGELLAN/MUSIC trial (NCT06815289) is further investigating MR-informed online adaptive SBRT for ultra-central lung tumors, with feasibility as the primary endpoint. In parallel, several studies are exploring the integration of SBRT with immune checkpoint inhibitors in this setting, including PACIFIC-4 (NCT03833154), ASTEROID trial (MEDI4736), and even though the KEYNOTE-867 trial, which used pembrolizumab plus SBRT, failed to improve the primary endpoint of event-free survival and OS compared to placebo plus SBRT [30].

Following the landmark SABR-COMET study, the management of oligometastatic disease has evolved to incorporate SBRT as a metastasis-directed treatment strategy [31]. Pulmonary metastases represent one of the most common sites of dissemination for many solid malignancies, and in the oligometastatic setting with lung involvement, the addition of definitive local therapy to standard systemic treatment has been shown to improve both PFS and OS [31,32,33]. A recent meta-analysis focusing on SBRT for pulmonary metastases further demonstrated high local control rates with a low risk of radiation-induced toxicity, underscoring the relevance of safe SBRT delivery to lung lesions, including those in central and ultra-central locations [34]. Furthermore, in the oligometastatic NSCLC, integrating SBRT alone or combined with immunotherapy or driver-mutated therapies is an active scientific field with promising outcomes [35,36,37,38,39].

The strengths of this study include the use of uniform SBRT planning principles derived from a landmark prospective protocol, centralized follow-up with mature outcome data, and systematic documentation of achieved dosimetric parameters for OARs. Several limitations of this study should be acknowledged. First, the retrospective design and the relatively limited sample size, particularly within the ultra-central subgroup (n = 26), restrict the ability to perform robust multivariable analyses and may limit the generalizability of the findings. As a result, comparisons between central and ultra-central tumors should be interpreted as exploratory and hypothesis-generating rather than definitive. In addition, multiple subgroup comparisons were performed, and given the limited number of events, these analyses should be interpreted cautiously. Histologic subtype distribution also differed between central and ultra-central groups, with a higher proportion of squamous cell carcinoma among ultra-central tumors; the limited number of events precluded meaningful adjustment for histology, and its potential influence on outcomes cannot be excluded. Second, although treatment delivery occurred across more than one radiotherapy center, follow-up and outcome assessment were centralized at a single academic institution, which may reflect institutional expertise and careful patient selection. Furthermore, the retrospective nature of the study introduces the possibility of selection bias in treatment allocation, referral bias toward specialized centers, and information bias due to non-blinded toxicity assessment. While achieved dosimetric parameters for organs at risk were systematically documented and analyzed, detailed target-related dosimetric metrics (e.g., PTV coverage, conformity, heterogeneity, or D2cm) were not systematically archived in the centralized follow-up dataset and therefore could not be analyzed. Consequently, we could not evaluate the extent to which PTV coverage may have been compromised to respect organ-at-risk constraints. In addition, because the treatment platform was closely linked to tumor location (EDGE for all central tumors and CyberKnife mainly for ultra-central tumors), platform-specific outcome comparisons were not feasible, representing a limitation of this retrospective analysis. Finally, radiographic response assessment following SBRT remains inherently challenging due to post-treatment inflammatory and fibrotic changes, despite multidisciplinary review and histological confirmation when feasible. In a minority of patients, histological confirmation was not feasible due to comorbidities or procedural risk, and diagnosis was based on multidisciplinary consensus and imaging findings, reflecting real-world practice in medically inoperable patients. The use of RECIST 1.1 criteria in this setting may lead to misclassification of post-treatment fibrosis or inflammatory changes as tumor progression, as imaging findings after SBRT do not always correlate with viable tumor. Although multidisciplinary review and histological confirmation were performed when clinically feasible, many cases were necessarily evaluated based on serial imaging alone, which represents an inherent limitation of imaging-based response assessment after SBRT. This adjudication approach, while clinically appropriate, may introduce assessment bias because histological reassessment was not uniformly feasible, and some cases relied on imaging-based consensus rather than pathological confirmation.

In summary, these real-world data support the safe and effective use of SBRT for carefully selected patients with centrally and proximity-defined ultra-centrally located early-stage lung tumors when rigorous dose-fractionation, planning, and organ-at-risk constraints derived from prospective protocols are applied. These findings reflect outcomes achieved in experienced centers using conservative, constraint-driven planning and may not be generalizable to overlap-defined ultra-central tumors or higher-risk clinical scenarios.

## 5. Conclusions

In this real-world, multi-center cohort with centralized follow-up, SBRT delivered according to dose-fractionation and planning principles derived from the NRG Oncology/RTOG 0813 protocol achieved excellent local control with minimal clinically relevant toxicity in patients with central and proximity-defined ultra-central early-stage NSCLC. These findings support the safe implementation of SBRT for carefully selected patients in routine clinical practice when stringent organ-at-risk constraints and conservative treatment planning are applied. Ongoing prospective studies, including trials such as STRICT/STAR-LUNG, are expected to further refine patient selection and optimize treatment strategies, particularly for ultra-central disease.

## Figures and Tables

**Table 1:** RTOG 0813 SBRT plan quality assurance checklist.

Category	Structure/Metric	Dosimetric Parameter	Constraint (RTOG 0813)
Normalization	PTV	Dose at COM_PTV	100% (normalization point)
Target Coverage	PTV	V100%	≥95%
Target Coverage	PTV	V90%	≥99%
Heterogeneity	PTV	Prescription isodose level	60–90% of COM_PTV dose
High-dose spillage	Body–PTV	V >105% Rx outside PTV	≤15% of PTV
Conformality	PTV	R100% (PIV/PTV)	<1.2 (≤1.5 acceptable)
Low-dose spillage	2 cm ring from PTV	D2 cm	≤protocol table (PTV-size based)
OAR	Total lung–GTV	V20Gy	≤10% (≤15% variation)
OAR	Total lung–GTV	D1500 cc	≤12.5 Gy
OAR	Total lung–GTV	D1000 cc	≤13.5 Gy
OAR	Spinal cord	D0.25 cc	≤22.5 Gy
OAR	Spinal cord	Dmax	≤30 Gy
OAR	Esophagus	D5 cc	≤27.5 Gy
OAR	Esophagus	Dmax	≤105% Rx
OAR	Trachea/Bronchial tree	D4 cc	≤18 Gy
OAR	Heart/Pericardium	D15 cc	≤32 Gy
OAR	Great vessels	D10 cc	≤47 Gy
OAR	Brachial plexus	D3 cc	≤30 Gy
OAR	Skin	D10 cc	≤30 Gy

Abbreviations: SBRT, stereotactic body radiotherapy; PTV, planning target volume; GTV, gross tumor volume; COM, center of mass; Rx, prescription dose; PIV, prescription isodose volume; OAR, organ at risk.

**Table 2:** Patients’ characteristics.

	Overall	Central	Ultra-Central	* *p*-Value
Number of patients	78	52	26	
Age, years—median (IQR)	76.0 (69.0–80.0)	76.0 (69.0–80.0)	76.0 (68.0–77.5)	0.146
Male	39 (50.0%)	32 (61.5%)	7 (26.9%)	0.008
Female	39 (50.0%)	20 (38.5%)	19 (73.1%)	
ECOG PS				0.022
0	27 (34.6%)	13 (25.0%)	14 (53.8%)	
≥1	51 (65.4%)	39 (75.0%)	12 (46.2%)	
Histology				0.028
Adenocarcinoma	24 (30.8%)	15 (28.8%)	9 (34.6%)	
Squamous	42 (53.8%)	25 (48.1%)	17 (65.4%)	
No biopsy	12 (15.4%)	12 (23.1%)	0 (0.0%)	
Tumor size, mm—median (IQR)	17.0 (16.2–20.0)	17.0 (16.0–20.0)	17.0 (17.0–18.0)	0.307
Tumor volume, cc—median (IQR)	23.2 (21.4–24.1)	23.2 (22.1–26.1)	22.2 (7.5–23.5)	0.004
cT stage (AJCC 8th)				0.482
T1a–T1b (≤2 cm)	68 (87.2%)	44 (84.6%)	24 (92.3%)	
T1c	10 (12.8%)	8 (15.4%)	2 (7.7%)	
Tumor lobe location				0.449
RML	19 (24.4%)	11 (21.2%)	8 (30.8%)	
RLL	22 (28.2%)	13 (25.0%)	9 (34.6%)	
LUL	18 (23.1%)	14 (26.9%)	4 (15.4%)	
LLL	19 (24.4%)	14 (26.9%)	5 (19.2%)	
EDGE	64 (82.1%)	52 (100%)	12 (46.2%)	<0.001
CyberKnife	14 (17.9%)	0 (0%)	14 (53.8%)	

* *p*-values refer to comparisons between patients with central and ultra-central tumors. Abbreviations: IQR, interquartile range; ECOG, Eastern Cooperative Oncology Group; PS, performance status; AJCC, American Joint Committee on Cancer; RML, right middle lobe; RLL, right lower lobe; LUL, left upper lobe; LLL, left lower lobe.

**Table 3:** Tumor response according to RECIST criteria.

Response Category (RECIST)	Overall Cohort	Central Cohort	Ultra-Central Cohort
CR	38 (48.7%)	26 (50.0%)	12 (46.2%)
PR	34 (43.6%)	22 (42.3%)	12 (46.2%)
SD	1 (1.3%)	0 (0.0%)	1 (3.8%)
PD	5 (6.4%)	4 (7.7%)	1 (3.8%)
ORR	72 (92.3%)	48 (92.3%)	24 (92.3%)

Abbreviations: RECIST, Response Evaluation Criteria in Solid Tumors; CR, complete response; PR, partial response; SD, stable disease; PD, progressing disease; ORR, objective response rate.

**Table 4:** Treatment-related toxicity (maximum CTCAE grade).

Toxicity	Overall (n = 78)	Central (n = 52)	Ultra-Central (n = 26)
Any toxicity (grade ≥ 1)	8 (10.3%)	5 (9.6%)	3 (11.5%)
Grade 1	7 (9.0%)	5 (9.6%)	2 (7.7%)
Grade 2	1 (1.3%)	0 (0.0%)	1 (3.8%)
Grade ≥ 3	0 (0.0%)	0 (0.0%)	0 (0.0%)
Clinically relevant toxicity (grade ≥ 2)	1 (1.3%)	0 (0.0%)	1 (3.8%)
Late toxicity (yes)	3 (3.8%)	0 (0.0%)	3 (11.5%)

Footnote: Data are presented as n (%). Clinically relevant toxicity was defined as CTCAE grade ≥ 2. Abbreviations: CTCAE, Common Terminology Criteria for Adverse Events.

**Table 5:** Achieved dosimetric parameters for organs at risk compared with NRG Oncology/RTOG 0813 constraints.

Organ at Risk	Dosimetric Parameter	RTOG 0813 Planning Constraint	Achieved Dosimetric Outcome (This Study)
Lung (total lung–GTV)	D1500cc	≤12.5 Gy	≤12.5 Gy (all patients)
Lung (total lung–GTV)	D1000cc	≤13.5 Gy	≤13.5 Gy (all patients)
Lung (total lung–GTV)	V20Gy	≤10% (≤15% variation)	≤10% in all evaluable plans
Spinal cord	Dmax	≤30 Gy	<30 Gy
Spinal cord	D0.25cc	≤22.5 Gy	≤23 Gy (D0.35cc)
Esophagus	Dmax	≤35 Gy	<35 Gy
Esophagus	D5cc	≤27.5 Gy	≤19.5 Gy
Trachea	D4cc	≤18 Gy	≤16.5 Gy
Proximal bronchial tree	D0.5cc	≤21 Gy	≤21 Gy
Proximal bronchial tree	Dmax	≤33 Gy	<33 Gy
Heart/Pericardium	D15cc	≤32 Gy	≤32 Gy
Great vessels	D10cc	≤47 Gy	≤47 Gy

Footnote: Dose–volume constraints were defined according to the NRG Oncology/RTOG 0813 protocol and applied during treatment planning. The values presented represent achieved dosimetric outcomes across the cohort. Abbreviations: GTV, gross tumor volume; Dmax, maximum dose; Dxcc, dose to x cubic centimeters; V20Gy, percentage of lung volume receiving ≥ 20 Gy.

## Data Availability

The data presented in this study are available on request from the corresponding author due to privacy and ethical reasons.

## Supplementary Materials

The following supporting information can be downloaded at: https://www.mdpi.com/article/10.3390/cancers18040653/s1. Figure S1. LPFS of the overall cohort; Figure S2. PFS of the overall cohort; Figure S3. OS for the entire cohort; Table S1. Definitions of central and ultra-central lung tumors across key studies.

## Abbreviations

The following abbreviations are used in this manuscript:

NSCLC	Non-Small Cell Lung Cancer
CTCAE	Common Terminology Criteria for Adverse Events
PET/CT	Positron Emission Tomography/Computed Tomography
SBRT	Stereotactic Body Radiotherapy
4D-CT	Four-Dimensional Computed Tomography
IGRT	Image-Guided Radiotherapy
CBCT	Cone-Beam Computed Tomography
SGRT	Surface-Guided Radiotherapy
LPFS	Local Progression-Free Survival
GTV	Gross Tumor Volume
CTV	Clinical Target Volume
ITV	Internal Target Volume
PTV	Planning Target Volume
BED	Biologically Effective Dose
OAR	Organ at Risk
PFS	Progression-Free Survival
ORR	Objective Response Rate
OS	Overall Survival
CT	Computed Tomography
