# Supplementary material for: Real-World Data on the Safety and Efficacy of SBRT for Central and Ultra-Central Lung Tumors: A Retrospective Multi-Center Cohort
Source: Cancers (Basel). 2026 Feb 17;18(4):653. doi: 10.3390/cancers18040653 (PMC12939535; doi:10.3390/cancers18040653)
Supplement: Supplementary file 1 [file cancers-18-00653-s001.zip › cancers-4125767-supplementary.pdf]

Supplementary material

Figure S1. Kaplan–Meier estimate of local progression-free survival (LPFS) for the entire study cohort. LPFS was defined as the time from the first day of SBRT to radiographic local tumor progression within the irradiated planning target volume or death from any cause.

Figure S1.LPFS of overall cohort.

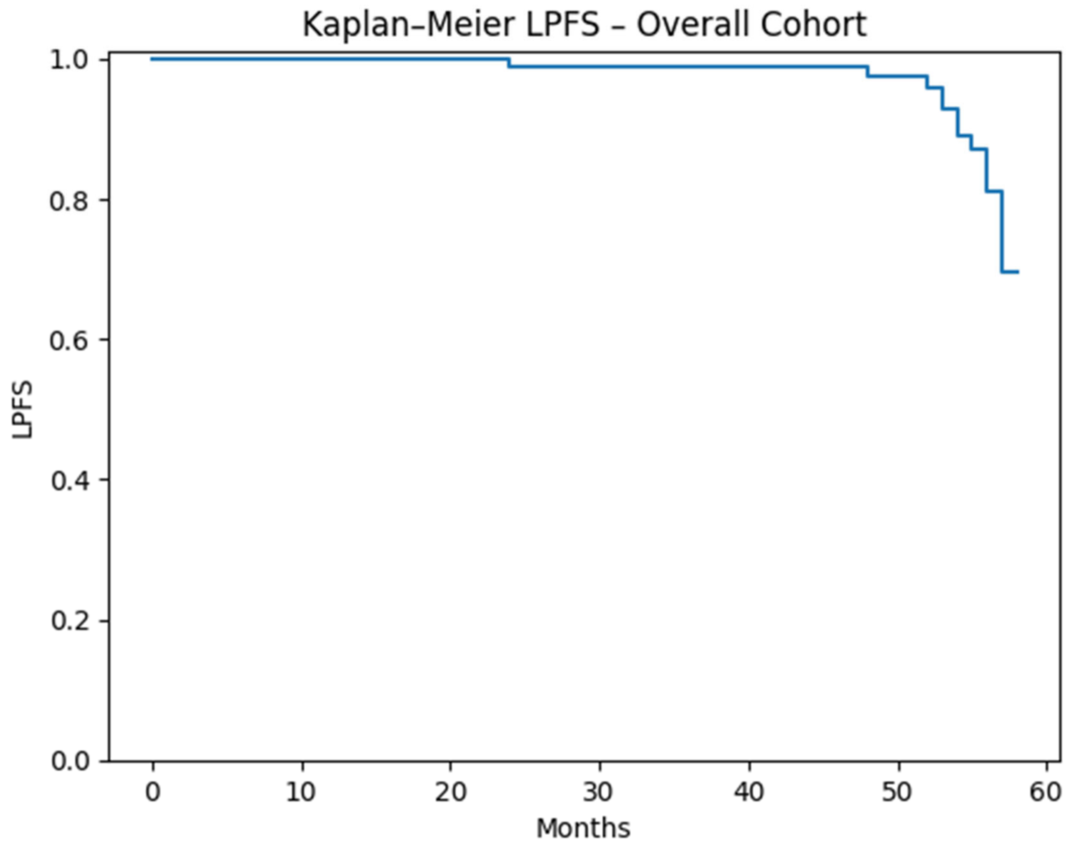

Figure S2. Kaplan–Meier estimate of progression-free survival (PFS) for the entire study cohort. PFS was defined as the time from the first day of SBRT to disease progression (local, regional, or distant) or death from any cause. Radiographic progression that was histologically reassessed and demonstrated no viable tumor was not considered a progression event. The analysis was administratively truncated at 60 months.

Figure S2. PFS of overall cohort.

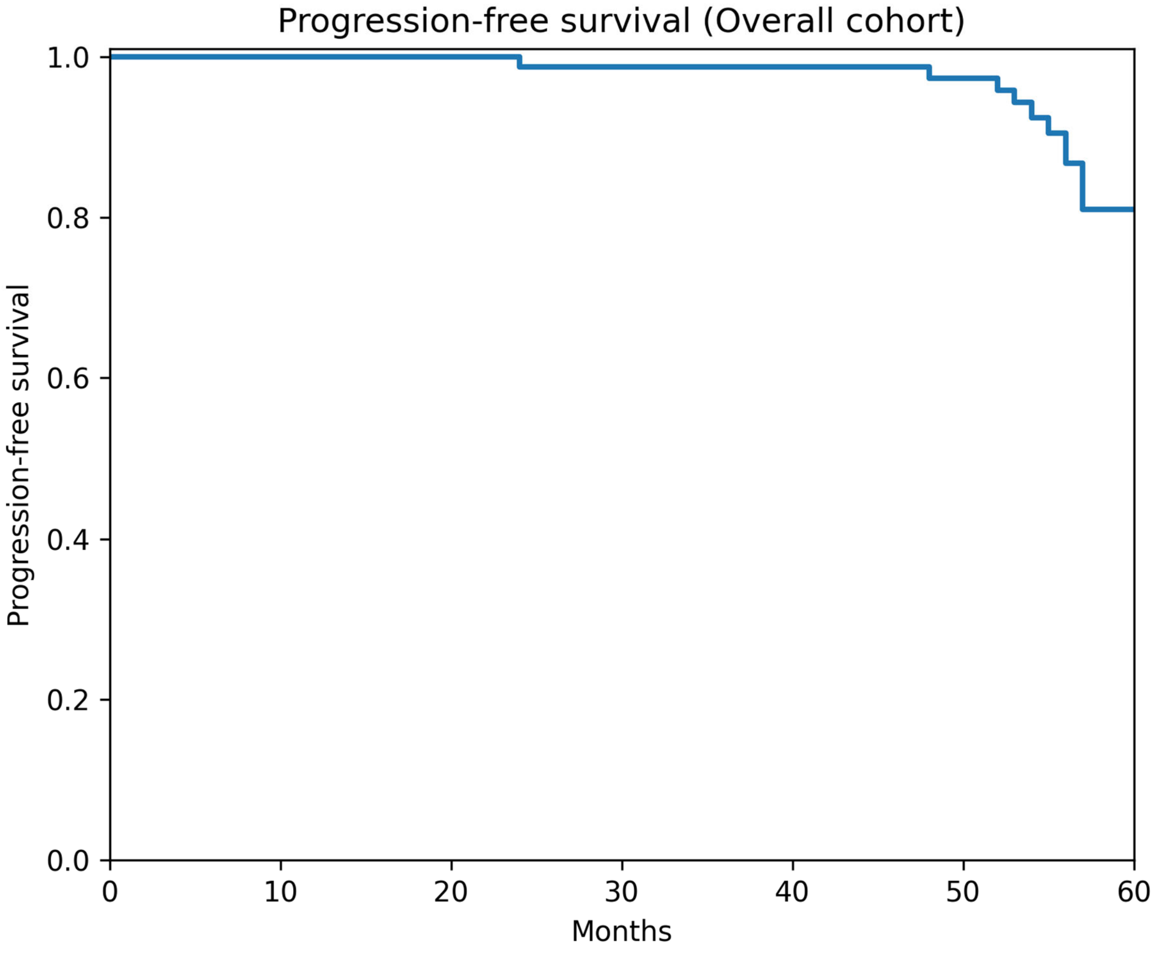

Figure S3. Kaplan–Meier estimate of overall survival (OS) for the entire study cohort. OS was calculated from the first day of SBRT to death from any cause. The analysis was administratively truncated at 60 months.

Figure S3. OS for the entire cohort.

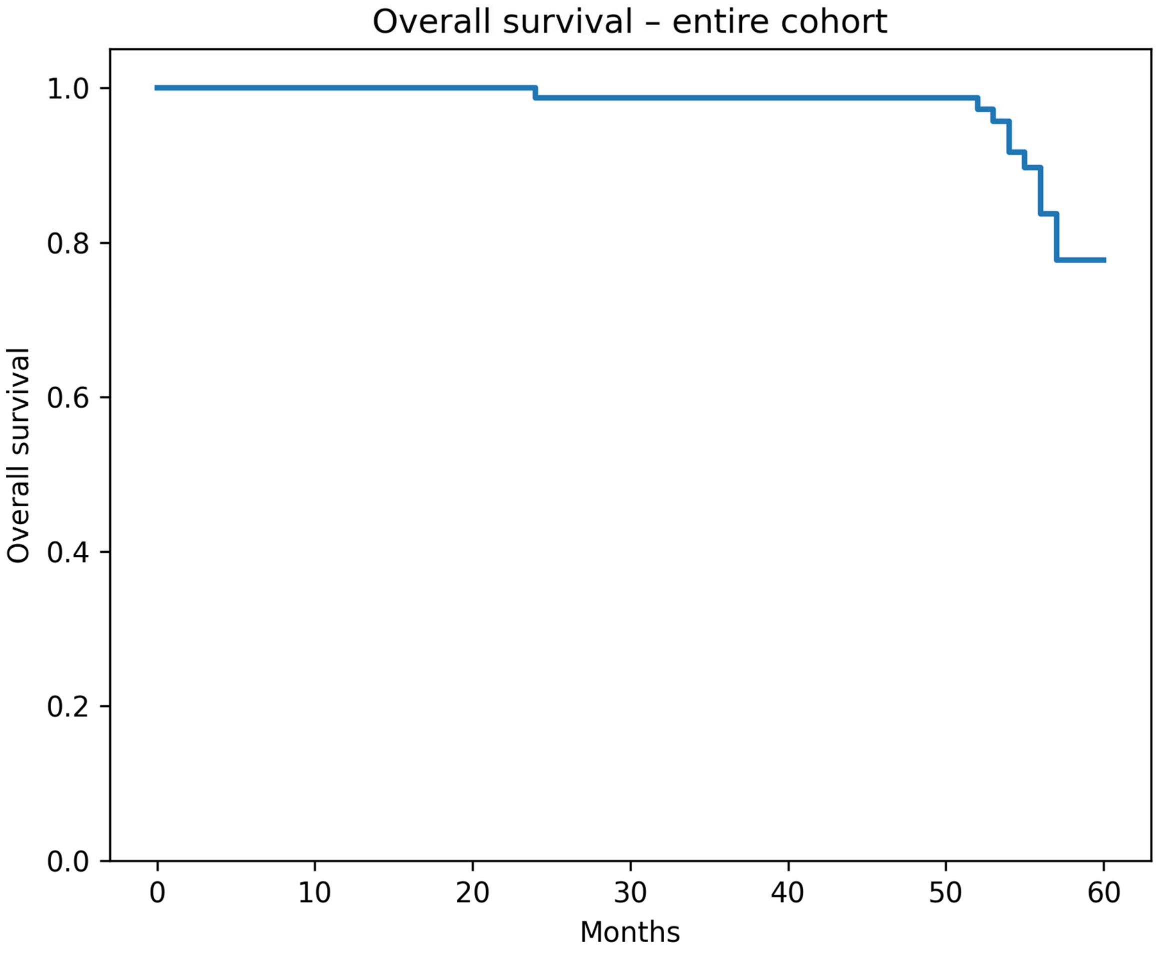

Supplementary Table S1. Definitions of central and ultra-central lung tumors across key studies.

Study / guideline	"Central" definition	"Ultra-central" / highest-risk definition	Key implication for toxicity interpretation
**IASLC (central tumors)**	GTV located within a 2 cm zone around mediastinal critical structures, including the bronchial tree, major vessels, heart, esophagus, spinal cord, phrenic nerve, recurrent laryngeal nerve, and brachial plexus	Not specifically defined	Central tumors are heterogeneous; toxicity risk increases with closer proximity or overlap with critical mediastinal structures
**HILUS trial**	Central tumors near mediastinal structures	"Ultra-central" proximity-based: tumor located ≤1 cm from the trachea or mainstem bronchi	Proximity-based ultra-central tumors include lesions near critical airways but may exclude direct overlap; toxicity risk generally lower than overlap-defined cohorts
**SUNSET trial**	Central tumors considered high-risk; study focused on ultra-central disease	Overlap-based: PTV directly overlapping the proximal bronchial tree and/or trachea	Overlap-defined ultra-central tumors represent a higher-risk subgroup with increased probability of severe toxicity
**Present study**	IASLC-based definition: GTV within 2 cm of mediastinal critical structures	Ultra-central proximity-based: tumor ≤1 cm from the trachea or mainstem bronchus (aligned with HILUS)	Findings apply primarily to proximity-defined ultra-central tumors treated with strict OAR constraints; extrapolation to overlap-defined ultra-central disease should be cautious

Abbreviations: IASLC, International Association for the Study of Lung Cancer; GTV, gross tumor volume; PTV, planning target volume; OAR, organ at risk.
